# Medical History of Elderly Patients in the Emergency Setting: Not an Easy Point-of-Care Diagnostic Marker

**DOI:** 10.1155/2015/490947

**Published:** 2015-09-03

**Authors:** Tobias Lindner, Anna Slagman, Arthur Senkin, Martin Möckel, Julia Searle

**Affiliations:** Division of Emergency Medicine, Charité-Universitätsmedizin Berlin, Campus Virchow Klinikum (CVK), Augustenburger Platz 1, 13353 Berlin, Germany

## Abstract

*Background*. Medical histories are a crucially important diagnostic tool. Elderly patients represent a large and increasing group of emergency patients. Due to cognitive deficits, taking a reliable medical history in this patient group can be difficult. We sought to evaluate the medical history-taking in emergency patients above 75 years of age with respect to duration and completeness. *Methods*. Anonymous data of consecutive patients were recorded. Times for the defined basic medical history-taking were documented, as were the availability of other sources and times to assess these. *Results*. Data of 104 patients were included in the analysis. In a quarter of patients (25%, *n* = 26) no complete basic medical history could be obtained. In the group of patients where complete data could be gathered, only 16 patients were able to provide all necessary information on their own. Including other sources like relatives or GPs prolonged the time until complete medical history from 7.3 minutes (patient only) to 26.4 (+relatives) and 56.3 (+GP) minutes. *Conclusions*. Medical histories are important diagnostic tools in the emergency setting and are prolonged in the elderly, especially if additional documentation and third parties need to be involved. New technologies like emergency medical cards might help to improve the availability of important patient data but implementation of these technologies is costly and faces data protection issues.

## 1. Introduction

Life-threatening emergencies in the preclinical and clinical setting often require a symptom-based approach, especially if information on the patient's medical history is unreliable or lacking. Information on preexisting diseases, medication, allergies, and incompatibilities is crucially important for a suitable diagnostic and therapeutic strategy. Unfortunately, due to either the specific emergency setting and/or due to patient-related factors like impaired vigilance, cognitive deficiencies, and psychological stress, evaluating a medical history can be difficult or even impossible [1]. Even for patients who are electively transferred for hospitalization by their general practitioner, information on the medical history can be very sparse, because the transfer process is not standardized. Likewise, information accompanying patients who are transferred from nursing homes or the outpatient sector is of very varying quality [2–4]. Often, the treating physician in the emergency department is reliant on medication packaging, medication lists of unknown date, or loose papers which are found amongst the patient's belongings.

A lack of knowledge regarding concomitant diseases including organ failure, medication [4–6], or allergies can cause unsafe, inadequate, delayed, or prolonged treatment [7]. For example, recent adjustment of a patient's medication might explain his/her symptoms, prevent potentially harmful measures [8], and enable a simple and cost-effective treatment strategy. Likewise, information on concomitant diseases, the availability of recent laboratory results, and the knowledge of, for example, an allergy to contrast media can help to speed up emergency management processes, avoid unnecessarily duplicated diagnostic testing, and improve risk stratification.

Assessment of a patient's medical history potentially takes up a considerable amount of time in the acute setting, where time is often sparse. Particularly in elderly (and often multimorbid) patients with cognitive deficiencies, taking a medical history can be time consuming and information is potentially unreliable.

We sought to investigate the physician-related time effort of assessing a standard medical history in patients with an age above 75 years presenting to the trauma unit in the emergency department of a tertiary care hospital. Secondary objectives were to evaluate how often a complete medical history can be obtained in these patients, to which extent information can be provided by the patient themselves, and what other measures were necessary to retrieve relevant information.

## 2. Patients and Methods

We performed a prospective cohort study in the trauma unit of our emergency department at Campus Virchow Klinikum of the Charité Berlin, Germany. The unit cares for around 50.000 cases of predominantly traumatologic and orthopedic origin. The study complies with the Declaration of Helsinki and was approved by the ethics committee of our institution (Ethikkommission Charité Berlin EA1/367/13). All data were attained by medical students doing their internship in the emergency department during routine patient management and were documented anonymously into a case report form.

From August 2013 to January 2014, data on men and women ≥75 years of age who presented to the trauma unit were reported by 5 medical students in 5 periods of 2 weeks each. For convenience reasons data were recorded if patients presented on workdays between 9 a.m. and 4 p.m. Students were asked to constantly check the central screen in the emergency department on which patients appear after triage and the administrative procedure in order to be seen by a physician. Among other things, details on the monitor contain the exact age of the patients, so that students could identify the group ≥75 years and immediately enroll them. Patients with life-threatening emergencies or in severe pain were excluded (assessment by physician on-call).

Documentation of the basic medical history included current medication, known diseases, allergies, and name and address of the general practitioner. Additionally, the students documented whether and to which extend these data could be obtained from the patient. Questions were open without suggestion of possible answers. The time needed for obtaining the available information was also recorded. In patients who could provide no or incomplete information, written medical documentation provided by the patient, especially medication lists, and an assessment of when these had been created or updated were sought.

If any written documentation was available, the time it took to look through and gather the necessary information from these documents was recorded.

In cases of doubt regarding the information provided by the patient or his/her written documents, other sources were sought, for example, relatives or the patient's GP. Again, the time it took to contact these sources and to retrieve the necessary information was recorded. The time needed to be invested by a physician was recorded only if it led to successful retrieval of the medical history.

All documentation was carried out on a prepared one-sided examination sheet. This sheet was blinded and also exact age and gender were not documented with respect to data protection issues and avoidance of the possibility of reidentification.

*Statistics*. Data were analyzed using SPSS software (IBM SPSS Statistics, Version 19). Nominal variables are shown as absolute and relative frequencies; categorical variables are shown as median with interquartile ranges (IQR), as well as minimum and maximum values.

## 3. Results

We recorded data on a total of 104 patients with an age of 75 years or above. The majority of patients came to the ED from their home (*n* = 72; 69.2%) or were brought in from a nursing home (*n* = 24; 23.1%). Two patients (1.9%) were brought in from a public place (street), another two were brought in from a GP (1.9%), and one was brought in from a rehabilitation clinic. In three cases (2.9%) documentation of the patients origin was left blank.

### 3.1. Availability of Basic Medical History Information

We differentiated between two groups, with regard to the availability of a complete basic medical information (defined as complete information about current medication, preknown diseases, allergies, and general practitioner).

#### 3.1.1. Group 1: No Complete Basic Medical Data Achievable

**Figure 1:**
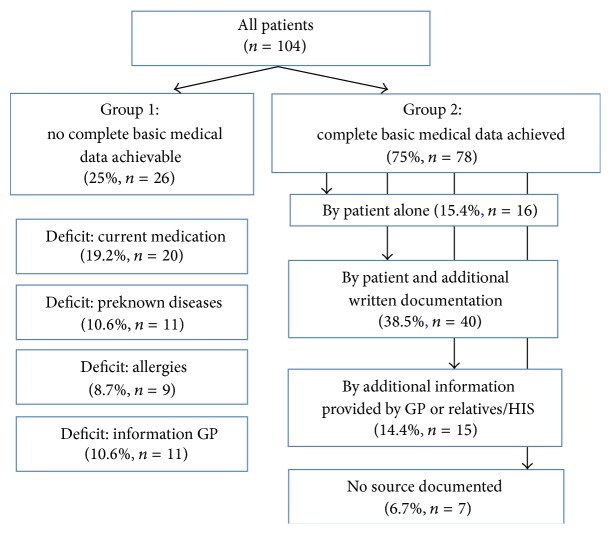
Distribution of patients included (Groups 1 and 2) (total number and % of all patients included): Group 1: no complete basic medical data achieved: single item deficit. Group 2: complete basic medical data achieved: by what means.

A total of 25% (*n* = 26) of the patients included had to be treated without the knowledge of their complete basic medical history (current medication, preknown diseases, allergies, and GP) (Figure 1), although 12 of these patients (46.2%) were carrying medical reports with them.

*Current Medication*. 25 patients (24%) in this group could not give appropriate answer to a current medication actively by themselves. 11 medical reports contained a list of medications (one set of data concerning eventual existence of list of medications missing), but only five of these lists were recognizably not older than 3 months, so that these five patients at least could ensure sufficient evidence about the drugs they currently take, leaving 20 patients (19.2%) without adequate information on this item (Figure 1). 

*Preknown Diseases.* 19 patients (18.3%) could not give appropriate answer to their preexisting illnesses actively by themselves. Medical reports of eight of these patients contained a list of preknown diseases (one set of data concerning eventual notification of preknown diseases in medical record missing), leaving 11 patients (10.6%) without adequate information on this item (Figure 1).

*Allergies.* Ten patients (9.6%) could not provide safe information on eventually existing allergies actively on their own. Only in one medical record of these patients an allergy status was retrieved to help out, leaving nine patients (8.7%) without adequate information on this item (Figure 1).

*General Practitioner*. 15 patients (14.4%) could not give sufficient information on their GP themselves that enabled medical staff to contact those. In four medical records of this patient group this information was achievable (one set of data concerning information on GP in medical record missing), leaving 11 patients (10.6%) without adequate information on this item (Figure 1).

#### 3.1.2. Group 2: Complete Basic Medical Data Achieved

In 75% (*n* = 78) of all patients complete basic medical data as defined was achievable (Figure 1).

However, only 15.4% of all patients (*n* = 16) were able to provide the necessary information completely on their own without any support of documents or other sources.

In 38.5% (*n* = 40) complete data was obtained through the information given by the patient himself combined with written documentation the patient had brought with him to hospital. In 14.4% (*n* = 15) data had to be completed by a third party (GP or relatives) or the hospital information system (Figure 1).

#### 3.1.3. Time Resources for Taking the Complete Medical History

In patients where a complete medical history could be established no matter how (*n* = 78), this took a median time of 12 minutes (interquartile range (IQR) 8/16.25; minimum 3 min., maximum 240 min.). The active physician's engagement time was 6 (5/10) minutes in patients who could provide a full medical history and was prolonged to around 11 (8/15) minutes if medical documents needed to be looked at. In cases where the GP needed to be contacted, establishing the medical history took a median time of 20 (15/81) minutes with a wide range (minimum 15 minutes, maximum 240 minutes) (Table 1).

## 4. Discussion

Our data show how difficult and time consuming it is to establish a reliable and complete medical history in emergency patients above 75 years of age.

A quarter of the patients in our study had to be managed without complete information about current medication, preknown diseases, possible allergies, or a contact's information of their GP. In particular, the availability of up-to-date medication charts seems to be problematic as almost every fifth patient had to be treated without adequate information on this item. Only 15% were able to provide all the information without the help of other sources. In most cases a successful completion of medical history was achieved by combination of talking to the patient and reading the documents he brought with him. As was to be expected, the physician's engagement time was shortest for patients who could provide all data of their medical history themselves. In cases where the GP had to be contacted, completion of the medical history was prolonged to 20 minutes (median) with a maximum time of 240 minutes, although this included waiting times for return calls, faxes, and so forth, during which time the physician is not actively bound. According to the German Medical Council, establishing a patient's medical history is a nondelegable responsibility of the treating physician [9]. If the process of taking the medical history is being improved to make it more reliable (and thereby safer) and faster, emergency departments gain valuable physician resources needed to diagnose and treat the patient's acute condition.

### 4.1. Elderly Patients in the Emergency Department

Due to the demographic changes in western developed countries, emergency departments face an increasing number of elderly patients. A systematic review from Canada published in 2002 analyzed 11 international studies and found the ED attendance rate of elderly patients to be between 12 and 21%. The authors found a consistent overrepresentation rate in this age group as compared to the respective general population. Two of the included studies with a longitudinal design showed a constant increase of elderly patients within the previous decade [10]. From 2006 to 2011, ED visit rates in the US were consistently highest for patients aged 85 years and above (around 90 per 100.000 inhabitants) [11]. In our institution in 2013, a total of 2931 patients with an age ≥75 years were treated in the trauma unit of the emergency department, which equals 7.5% of all patients presenting to this unit. When extrapolating our data to these total numbers, 733 patients above the age of 75 years would have been cared for without knowledge of their basic medical history. With the information the patients were able to provide themselves alone, 1172 might have received medication without the cognition of possible allergies and 1465 might have received nonappropriate medication considering their preexisting diseases and possible pharmacological interactions.

Tambyln et al. recently published a study on the information gap between community-based pharmacy records and medication records in the ED of patients admitted to hospital [4]. They included 613 patients in two Canadian hospitals and found that the ED records did not list 41.5% of the medication documented in the community records. They concluded that there is a big need for health technologies that allow information transfer of current medication between hospital and community especially for elderly and multimorbid patients.

Morphet et al. recently published a retrospective study analyzing data of 408 resident transfers from aged care facilities to emergency departments in Australia in terms of transfer documentation [3]. Median age of transferred patients was 86 years. The authors defined seven essential sets of data for their quality control of information transfer to an ED. Amongst others, they also examined “past medical history,” “medication chart,” “allergy status,” and “GP contact details.” In 2007, Australian authorities had introduced the “transfer-to-hospital” envelope, which should contain patient's key clinical information for admission to an ED but also discharge information from hospital back into the outpatient system. Information on “past medical history” was present in 63%, on “medication” in 65%, on “allergies” in 56%, and on “GP details” in 69% of the 408 cases. In comparison to our data, this “envelope” seemed to improve information transfer but still is insufficient and it might be unreliable regarding updates and actuality.

### 4.2. Possible Solutions

To meet the requirements of emergency medicine to be safe and effective, new technologies for process optimization in emergency and acute care have been asked for in this issue and also in recent publications [2, 3, 5, 12, 13].

A possible solution to improve the currently unreliable and time consuming process of history-taking in the elderly could be offered by medical emergency cards that can provide updated and relevant patient information. A pilot survey by Olola et al. [14] in Utah evaluated the perceived usefulness of an emergency card and a continuity-of-care report in users. In the online survey amongst the 101 users (75,3% female, median age 56 years), 68% reported the card to be helpful, especially the emergency card. Users clearly prioritized up-to-date information emergency situations over information for routine visits. The same group evaluated what medical professionals (from outpatient clinics) thought about the usefulness of their card/report [15]. In accordance with the patients, 94% found the emergency medical card to be of help in medical decision making at the point of care, whereas only 74% were persuaded by the usefulness of the continuity-of-care report. However, with respect to decreasing time effort and increasing overall knowledge both documents were voted 100% helpful; regarding the influence on the process of decision making the agreement rate was 94% for both. The authors put emphasis on the fact that the stored information has to be accessible for and modifiable by the user, who should have a control function over his file.

In Germany, an electronic health card was introduced in 2009, but so far information attainable through this card is restricted to only basic data (name, date of birth, address, etc.). In a next step, it is planned to include an emergency data set, which should increase patient safety during emergency treatment and help to gain a more effective use of physician resources. However, there have been a lot of setbacks in the technical development of this card and there is still ongoing political discussion especially with regard to data protection issues. The World Medical Card (Bergen, Norway) (http://www.wmc-card.com/) is an already readily available product that covers all these aspects and stores patient-centered and patient-controlled medical and paramedical information for emergencies but also for routine visits. Knowing his own medical risk profile the user can individually weigh up the risk of disclosing relevant information for emergency situations against the risk that despite all efforts of data protection his data might become visible for persons he does not want to share this information with. It also serves as an organ donator card. Information transfer is ensured by a haptic card, on web (with an additional data storage for reports, X-rays, patient's provision, etc.) and on mobile devices. This emergency card was primarily introduced to assure information transfer for international travels. For communicating medical data it deploys international ATC-codes for medication and ICD.-10 codes for diseases as developed by the World Health Organization (WHO).

From a procedural point of view, however, even more extensive systems which also allow feed of approved data from a closed and patient-centered system like the World Medical Card directly into the hospital information system are favorable. This function in addition to its impact on medical history duration could potentially increase the time-saving aspect by avoiding noncurative activities like manually transcribing long lists of medication and concomitant diseases from a piece of paper into the hospital information system.

## 5. Limitations

Data were recorded anonymously and thus no data on age and gender are available. Patients were included if their age was above 75 years.

The quality of the medical history reported by the patients was not validated. Thus, the proportion of incomplete (or wrong) information might have been even higher.

We did not record any diagnoses or further treatment and we therefore have no data on disease severity. We also did not record whether an incomplete or prolonged medical history led to any delays in diagnostics or treatment. In our study, in 11.5% of patients basic data were only available through the patient's GP. We only included patients during working hours, where it should have potentially been easy to contact the patient's GP. It is to be expected that there are more cases with incomplete medical history outside these hours. Last, we did not record the time invested in the cases in which no basic medical history was achieved in the end. The medical histories in this study were taken and documented by medical students in their last year (PJ students/interns). Taking a medical history may be faster when this is done by an experienced physician.

## 6. Conclusion

Medical histories are important diagnostic tools in the emergency setting and are prolonged in the elderly, especially if additional documentation and third parties need to be involved. New technologies like emergency medical cards might help to improve the availability of important patient data but a general implementation of these technologies is costly and faces data protection issues.

Given the potentially high impact on the safety of emergency care in elderly patients, who present a large and increasing patient group in emergency departments, all efforts should be made to make these technologies available.

## Figures and Tables

**Table 1:** Sources of information and time until successfully completed basic medical history (*n* = 78).

Source(s) of information	Relative frequency in %	Duration [min.] Median (IQR) Minimum–maximum
Self-reported medical history only	15.4 (*n* = 16)	6 (5/10) 3–15
Self-reported medical history plus medical documentation	38.5 (*n* = 40)	11 (8/15) 4–26
Third-party medical history (GP)	8.7 (*n* = 9)	20 (15/81) 15–240
Third-party medical history (relatives)	4.8 (*n* = 5)	19 (14/43) 10–62
Data from hospital information system	0.1 (*n* = 1)	16
*No source documented*	6.7 (*n* = 7)	14 (12/19) 11–25

IQR:  interquartile range.
